# Individual and community determinants of neonatal mortality in Ghana: a multilevel analysis

**DOI:** 10.1186/1471-2393-14-165

**Published:** 2014-05-12

**Authors:** Gbenga A Kayode, Evelyn Ansah, Irene Akua Agyepong, Mary Amoakoh-Coleman, Diederick E Grobbee, Kerstin Klipstein-Grobusch

**Affiliations:** 1 Julius Global Health, Julius Center for Health Sciences and Primary Care, University Medical Centre Utrecht, P.O. Box 85500, Utrecht, GA 3508, The Netherlands; 2 Ghana Health Service, Greater Accra Region, Accra, Ghana; 3 Ghana School of Public Health, University of Ghana, Legon, Accra, Ghana; 4 School of Public Health, Faculty of Health Sciences, University of the Witwatersrand, Johannesburg, South Africa

**Keywords:** Neonatal mortality, Individual factors, Community factors, Multilevel analysis, Ghana

## Abstract

**Background:**

Neonatal mortality is a global challenge; identification of individual and community determinants associated with it are important for targeted interventions. However in most low and middle income countries (LMICs) including Ghana this problem has not been adequately investigated as the impact of contextual factors remains undetermined despite their significant influence on under-five mortality and morbidity.

**Methods:**

Based on a modified conceptual framework for child survival, hierarchical modelling was deployed to examine about 6,900 women, aged 15 – 49 years (level 1), nested within 412 communities (level 2) in Ghana by analysing combined data of the 2003 and 2008 Ghana Demographic and Health Survey. The aim was to identify individual (maternal, paternal, neonatal, antenatal, delivery and postnatal) and community (socioeconomic disadvantage communities) determinants associated with neonatal mortality.

**Results:**

The results showed both individual and community characteristics to be associated with neonatal mortality. Infants of multiple-gestation [OR 5.30; P-value < 0.001; 95% CI 2.81 – 10.00], neonates with inadequate birth spacing [OR 3.47; P-value < 0.01; 95% CI 1.60 – 7.57] and low birth weight [OR 2.01; P-value < 0.01; 95% CI 1.23 – 3.30] had a lower chance of surviving the neonatal period. Similarly, infants of grand multiparous mothers [OR 2.59; P-value < 0.05; 95% CI 1.03 – 6.49] and non-breastfed infants [OR 142.31; P-value < 0.001; 95% CI 80.19 – 252.54] were more likely to die during neonatal life, whereas adequate utilization of antenatal, delivery and postnatal health services [OR 0.25; P-value < 0.001; 95% CI 0.13 – 0.46] reduced the likelihood of neonatal mortality. Dwelling in a neighbourhood with high socioeconomic deprivation was associated with increased neonatal mortality [OR 3.38; P-value < 0.01; 95% CI 1.42 – 8.04].

**Conclusion:**

Both individual and community characteristics show a marked impact on neonatal survival. Implementation of community-based interventions addressing basic education, poverty alleviation, women empowerment and infrastructural development and an increased focus on the continuum-of-care approach in healthcare service will improve neonatal survival.

## Background

The first 28 days of life remain the most critical period for an infant to survive during childhood; [1] approximately 10,000 newborns die everyday during this period [2]. As a result of the devastating effects of childhood mortality especially in low and middle income countries (LMICs), 189 United Nations member states unanimously agreed to adopt reduction of under-five mortality by two-thirds between 1990 and 2015 as the Millennium Development Goal 4 (MDG 4) [3]. The deadline for the attainment of MDG 4 target is fast approaching. Yet up to 40 % of under-five mortality occur at neonatal stage even though two-thirds of these deaths are preventable [4]. Worldwide about three million newborns are dying annually [5] before attaining the age of one month and despite repeated “calls for action”, [1,2,6,7] this serious public health issue has not received desirable attention [8]. Consequently, in the last two decades, neonatal mortality has shown limited decline globally and in Sub-Saharan Africa (SSA) [1,9]. For instance in 2008 this region only witnessed a 2% decline in neonatal mortality [10]. Low birth weight, prematurity, infections, birth asphyxia and birth trauma have been identified as the leading causes of neonatal deaths worldwide [4], similar to the major causes of neonatal deaths in SSA [11] and Ghana [12-15]. Across the globe, there are great variations in neonatal mortality: 99% of neonatal deaths occur in LMICs [1], whereas until recently 99% of neonatal research publications were conducted in high income countries (HICs). This indicates a gross lack of information and knowledge of neonatal mortality in LMICs.

In Ghana, neonatal mortality is an important public health issue; 30 per 1000 live births are dying within the first 28 days of life [16]. In order to attain MDG 4 neonatal mortality has to reduce substantially because it accounts for more than half of the infant and under-five mortality [16]. Most studies to date mainly examined factors influencing under-five and infant mortality in LMICs [17], whereas only a limited number of studies have specifically identified factors associated with neonatal mortality in SSA. Early initiation of breastfeeding was shown to be inversely associated with neonatal mortality in Ghana [18]. Further in LMICs, neonatal (low birth weight, male infant, multiple pregnancy and prematurity) [19-21], maternal (single, nulliparous mothers and short birth spacing) [19-21], and health service factors (delivery and postnatal services) were reported to have independent associations with neonatal mortality [19,20].

These studies focused on the associations between individual-level factors and neonatal mortality. They typically did not disentangle the influence of individual and community determinants on neonatal mortality even when they analysed population-based data with hierarchical nature. In other words, most of these prior studies disregarded the importance of contextual phenomena because community-level determinants were not appropriately considered in their analyses. Contextual phenomenon is an intuitive core notion of social epidemiology; resting on the observation that people dwelling in the same neighbourhood tend to resemble each other in terms of their health outcomes more than those living in different areas. Thus, taking contextual factors into account either at the design and/or analytical phase is crucial in understanding individual health outcomes in a population.

In LMICs, neonatal mortality is yet to be adequately examined by multilevel analysis, an analytic method that has the capability of assessing both fixed and random effects in a single model. Application of multilevel analysis allows to disentangle the influence of individual and community characteristics on neonatal survival based on the level at which they shaped child survival. In contrast, the application of single-level analyses (individual or ecological analyses) instead of multilevel analyses will make it difficult to deduce whether community-level factors influence neonatal outcomes regardless of the individual characteristics or whether inter-community variation in neonatal mortality is exclusively due to their individual characteristics without any influence of community-level factors.

In addition, there is increasing evidence of associations between community-level factors and under-five stunting and mortality after considering individual factors [22-24]. The present study aims to identify both individual (biological or proximate) and community (contextual, societal or distal) factors associated with neonatal mortality in Ghana by examining Ghana Demographic and Health Survey (GDHS) data using hierarchical modelling.

## Methods

### Study design

This is a population-based study which examined the combined dataset of the 2003 and 2008 Ghana Demographic and Health Survey to identify individual and community determinants influencing neonatal mortality in Ghana.

### Data collection

Comprehensive information on the sampling techniques and procedures applied for data collection in the Ghana Demographic and Health Survey have been published elsewhere [16,25]. In brief, all women and men in all the selected households, aged 15 to 49 and 15 to 59 respectively were interviewed with the aid of questionnaires (household, women’s and men’s questionnaires). The questionnaires covered information on socioeconomic, demographic and health indicators. Informed consent was obtained from all the participants before face-to-face interviews were conducted. Information was obtained on under-five deaths in the last five years in both occasions. In both surveys combined, 12,474 households, 11,045 women and 10,114 men were identified for interviews and response rates of 99%, 96% and 94% respectively were observed [16,25].

### Ethical consideration

Ethical approval to conduct DHS in Ghana was approved by the Ethics Committee of ICF Macro in Calverton, USA and the Ethics Committee, Ghana Health Service, Accra, Ghana. We obtained ethics approval for analysis of this data from the Ethics Committee of ICF Macro in Calverton, USA.

### Variables

#### Outcome variable

Neonatal mortality was defined during the data collection as the probability of dying within the first month of life.

### Determinants

**Figure 1:**
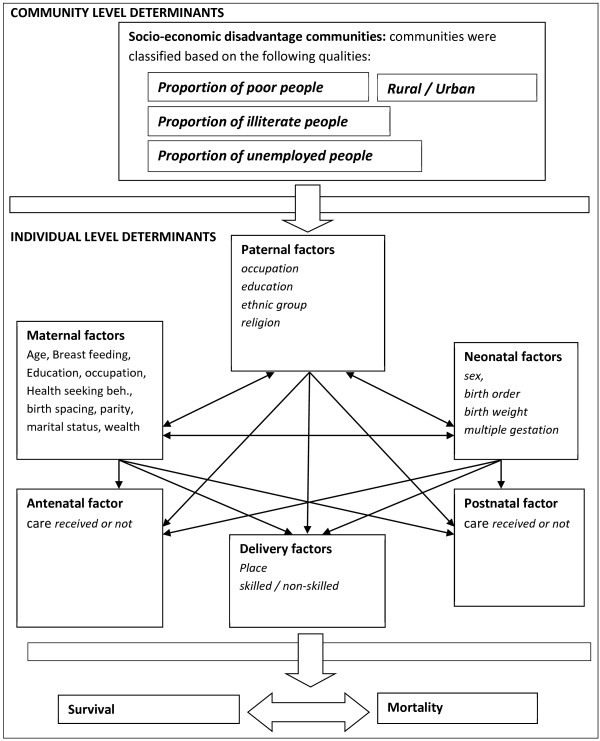
**Adapted version of the conceptual framework for individual & community-level determinants influencing neonatal mortality **[26]**.**

Individual and community characteristics that were examined for possible associations with neonatal mortality were based on an adapted framework of child survival [26] taking into account the available information in the 2003 and 2008 Ghana Demographic and Health Survey. The adapted framework for neonatal survival is depicted in Figure 1.

#### Individual-level determinants

Individual-level factors were categorized into six groups: maternal, neonatal, paternal, antenatal, delivery, and postnatal factors. Maternal factors encompassed maternal age, parity, maternal occupation, maternal education, breastfeeding and preceding birth interval. We examined sex (male/female), birth order, multiple pregnancy and birth weight to assess the effects of neonatal factors while paternal factors entailed paternal occupation and education, ethnic group and household wealth index. Mothers were asked whether the birth weight of their babies were very big, bigger than average, average, smaller than average or very small. We classified smaller birthweight than average and very small birthweight as small and average, bigger than average and very big as normal birthweight. Maternal uptake of antenatal, delivery and postnatal healthcare services were assessed by considering maternal health seeking behaviour. Maternal health seeking behaviour was operationalized by combining maternal characteristics such as having a health care card, having received tetanus toxoid, having received antenatal care, have delivered in a health facility and having knowledge of oral rehydration solution using Principal Component Analysis (PCA). To evaluate the wealth index of the households an asset based approach was applied by DHS. Household properties such as radio, car, and other features within the house such as water source, toilet facility and roof/floor type were utilized to evaluate the wealth index of the household using PCA [16,25]. Asset-based methods have previously been applied by the World Bank and other studies to estimate wealth status [27-29].

#### Community-level determinants

The community was used to represent the primary sampling unit (PSU) of the data. Community impact on neonatal mortality was assessed by considering the status of socioeconomic disadvantage of the community in which the participants were dwelling. Community socioeconomic disadvantage was operationalized by combining four factors: place of residence (rural/urban), and the proportion of illiteracy, unemployment and poverty (estimated asset index < 20% poorest quintile). PCA was applied to generate community socioeconomic disadvantage and subsequently classified into low, moderate and high deprivation tertiles. Communities with low socioeconomic disadvantage were the least deprived. A couple of studies have utilised community socioeconomic disadvantage as a community-level determinant [30-33].

### Statistical analyses

#### Descriptive analyses

Descriptive analysis was performed by evaluating the prevalence of neonatal mortality (outcome variable) across the categories of each explanatory variable. Also the frequency and percentage of each of the categories within the explanatory variables were obtained.

#### Modelling approaches

The hierarchical nature of the Ghana DHS data and the framework for neonatal survival were considered during the analysis. Thus, two-level multivariable multilevel logistic regression was applied. Individual-level determinants were nested within the community-level determinants in which they live. Three models were fitted in the analysis. Model 1 has no determinant variable (empty model). This was fitted to decompose the total variance between individual and community level. We included all the individual-level determinants into model 2 while the model 3 encompassed individual-level and community-level determinants.

#### Measures of association (fixed effects)

The effects of individual-level and community-level determinants on neonatal mortality were reported in term of odds ratios with their P-values and 95% confidence interval.

#### Measures of variation (random effects)

Random effects were expressed in terms of Intra-Cluster Correlation (ICC)/Variance Partition Coefficient (VPC) and Median Odds Ratio (MOR).

#### Model fitness & precision

The loglikelihood and Akaike Information Criterion (AIC) of the models were estimated to assess the fitness of the model relative to the other models. Variance Inflation Factor and Tolerance test were performed to identify the presence of multicollinearity in the model. StataSE 11 software package [34] was used for the analyses and statistical significance of the covariates were determined by two-tailed Wald test at significance level of alpha equal to 5%.

## Results

### Characteristics of the sample

The general characteristics of the study population are shown in Tables 1 and 2. Approximately 6,900 respondents living in 412 different communities were interviewed in the last decade in Ghana to obtain information on under-5 mortality. Half of the women interviewed were aged 25 to 34 years, 40% of them were illiterate, and about two-thirds of them engaged in manual labour jobs and were residing in rural settlements. More than half of the men were farmers even though more than two-thirds of them had at least a primary school education. About one-third of the communties were classified to be in abject poverty while 90% of the population were unemployed. About 3% of the newborns delivered were not breastfed, 17 % were having LBW and neonatal mortality accounted for more than half of under-five mortality and two-thirds of infant mortality. Neonatal deaths were observed to occur most often in newborns having birth spacing less than 18 months, low birth weight and those that were not breastfed. Similarly, for infants of multiple gestation and those in fifth or higher birth order prevalence of neonatal mortality was higher. Neonatal deaths were reported more often among infants of grand-multiparous women with poor health seeking behaviour. Further details are given in Tables 1 and 2. The time interval between the two subsequent data collection periods was not related to neonatal mortality; its rate has been more or less stationary in the last decade in Ghana [16,25].

**Table 1:** General characteristics of the study population: individual variables

		**Neonatal death**		
	**Number (%)**	**Yes**	**No**	
	**n (%)**	**n (%)**	**n (%)**	**Total N (%)**
**Individual-level determinants**				
**Neonatal factors**				
Infant sex				
*Male*	3,476 (51)	150 (4)	3,326 (96)	3,476 (100)
*Female*	3,360 (49)	122 (4)	3,238 (96)	3,360 (100)
Birth order				
*One*	1,518 (22)	66 (4)	1,452 (96)	1,518 (100)
*Two*	1,342 (20)	37 (3)	1,305 (97)	1,342 (100)
*Three*	1,090 (16)	37 (3)	1,053 (97)	1,090 (100)
*Four*	883 (13)	28 (3)	855 (97)	883 (100)
*Five*	2,003 (29)	104 (5)	1,899 (95)	2,003 (100)
Multiple gestation				
*Yes*	285 (4)	39 (14)	246 (86)	285 (100)
*No*	6,551 (96)	233 (4)	6,318 (96)	6,551 (100)
Birth weight				
*Small*	1,142 (17)	72 (6)	1,070 (94)	1,142 (100)
*Normal*	5,607 (83)	176 (3)	5,431 (97)	5,607 (100)
**Maternal factors**				
Maternal Age				
*15 – 24 years*	1,537 (23)	57 (4)	1,480 (96)	1,537 (100)
*25 – 34 years*	3,300 (48)	123 (4)	3,177 (96)	3,300 (100)
*35 – 49 years*	1,999 (29)	92 (5)	1,907 (95)	1,999 (100)
Maternal education				
*No education*	2,956 (43)	113 (4)	2,843 (96)	2,956 (100)
*Primary*	1,545 (23)	73 (5)	1,472 (95)	1,545 (100)
*Secondary or higher*	2,335 (34)	86 (4)	2,249 (96)	2,335 (100)
Maternal occupation				
*Unemployed*	689 (10)	27 (4)	662 (96)	689 (100)
*Manual*	4,184 (62)	167 (4)	4,017 (96)	4,184 (100)
*White collar job*	1,922 (28)	75 (4)	1,847 (96)	1,922 (100)
Parity				
*1*	1,035 (15)	27 (3)	1,008 (97)	1,035 (100)
*2 – 4*	3,514 (51)	148 (3)	3,396 (97)	3,514 (100)
*≥ 5*	2,287 (34)	127 (6)	2,160 (94)	2,287 (100)
Birth interval				
*< 18 months*	227 (4)	27 (12)	200 (88)	227 (100)
*18 – 36 months*	2,060 (39)	82 (4)	1,978 (96)	2,060 (100)
*> 36 months*	3,016 (57)	97 (3)	2,919 (97)	3,016 (100)
Breastfeeding				
*Yes*	6,647 (97)	138 (2)	6,509 (98)	6,647 (100)
*No*	189 (3)	134 (71)	55 (29)	77 (100)
Health seeking behaviour				
*1 lowest*	1,489 (24)	128 (9)	1,361 (91)	1,489 (100)
2	2,231 (35)	64 (3)	2,167 (97)	2,231 (100)
*3*	1,417 (22)	50 (4)	1,367 (96)	1,417 (100)
*4 Highest*	1,224 (19)	00 (0)	1,224 (100)	1,224 (100)
**Paternal factor**				
Paternal occupation				
*Farming*	3,816 (59)	146 (4)	3,670 (96)	3,816 (100)
*Manual*	1,481 (23)	52 (4)	1,429 (96)	1,481 (100)
*White collar job*	1,200 (18)	63 (5)	1,137 (95)	1,200 (100)
Paternal education				
*No education*	2,331 (38)	93 (4)	2,238 (95)	2,331 (100)
*Primary*	580 (9)	26 (4)	554 (96)	580 (100)
*Secondary or higher*	3,329 (53)	125 (4)	3,204 (96)	3,329 (100)
Wealth index				
*Poor*	3,773 (55)	136 (4)	3,637 (96)	3,773 (100)
*Middle*	1,186 (17)	61 (5)	1,125 (95)	1,186 (100)
*Rich*	1,877 (28)	75 (4)	1,802 (96)	1,877 (100)
Ethnicity				
Akan	2,612 (38)	110 (4)	2,502 (96)	2,612 (100)
*Ga/Guan*	578 (8)	21 (4)	557 (96)	578 (100)
*Ewe*	791 (12)	26 (3)	765 (97)	791 (100)
*Mole-dagbani*	1,697 (25)	62 (4)	1,635 (96)	1,697 (100)
*Grussi/Gruma*	703 (10)	35 (5)	668 (95)	703 (100)
*Others*	451 (7)	18 (4)	433 (96)	451 (100)

**Table 2:** General characteristics of the study population: community variables

		**Neonatal death**		
	**Number (%)**	**Yes**	**No**	
	**n (%)**	**n (%)**	**n (%)**	**Total N (%)**
**Community-level determinants**				
Place of residence				
*Rural*	4,793 (70)	198 (4)	4,595 (96)	4,793 (100)
*Urban*	2,043 (30)	74 (4)	1,969 (96)	2,043 (100)
Illiterate				
*No education*	3,880 (57)	159 (4)	3,721 (96)	3,880 (100)
*Educated*	2,956 (43)	113 (4)	2,843 (96)	2,956 (100)
Unemployment				
*Employed*	689 (10)	27 (4)	662 (96)	689 (100)
*Unemployed*	6,147 (90)	245 (4)	5,902 (96)	6,147 (100)
Poverty				
*lowest 20%*	2,174 (33)	84 (4)	2,174 (96)	2,258 (100)
*Above 20%*	4,578 (67)	188 (4)	4,390 (4)	4,578 (100)

### Random effects (Measure of variations)

Table 3 depicts the results of the variance component model which is also referred to as null model or empty model (model 1). This model was applied to estimate the total variance in neonatal mortality that can be attributed to the communities in which the mothers were living; in other words, community-level variance was estimated in order to justify the applicability of multivariable multilevel regression analysis (MMLRA). Community-level variance was statistically significant (P-value < 0.05); it showed that some of the total variance in neonatal mortality can be explained by community-level determinants thus MMLRA was performed to adequately consider community-level factors. The intracluster correlation/intra-community correlation (ICC) or variance partition coefficient (VPC) was estimated at 0.07 which simply means that 7% of the total variance in neonatal mortality in Ghana can be attributed to the communities in which the mothers were residing. This also implies that the correlation between mothers living in the same community regarding the likelihood of experiencing neonatal mortality was 0.07. The estimated community variance was also expressed as median odds ratios (MOR = 1.58) which means that the likelihood of having neonatal mortality increased by 58% when a woman moved from a community with lower risk to a higher risk community.

**Table 3:** Associations between neonatal mortality and individual and community level determinants

	**Null model**	**Mode with individual level determinants**	**Mode with individual & community level determinants**
**Fixed effect (OR, 95% CI, P-value)**			
**Individual-level determinants**			
Infant sex			
*Male*		1 (reference)	1 (reference)
*Female*		0.78 (0.51 – 1.19)	0.79 (0.52 – 1.21)
Birth order			
*One*		1 (reference)	1 (reference)
*Two*		---	---
*Three*		1.58 (0.79 – 3.17)	1.57 (0.78 – 3.16)
*Four*		0.81 (0.33 – 1.99)	0.77 (0.31 – 1.93)
*Five*		0.77 (0.25 – 2.42)	0.72 (0.23 – 2.28)
Multiple pregnancy			
*Yes*		5.30 (2.82 – 9.95)***	5.30 (2.81 – 10.00)***
*No*		1 (reference)	1 (reference)
Birth weight			
*Small (<2.5 kg)*		2.01 (1.23 – 3.29)**	2.01 (1.23 – 3.30)**
*Normal (≥2.5 kg)*		1 (reference)	1 (reference)
**Maternal factors**			
Maternal Age			
*15 – 24 years*		1 (reference)	1 (reference)
*25 – 34 years*		0.74 (0.34 – 1.59)	0.75 (0.34 – 1.64)
*35 – 49 years*		0.73 (0.29 – 1.80)	0.74 (0.30 – 1.87)
Maternal education			
*No education*		0.74 (0.36 – 1.54)	0.88 (0.42 – 1.83)
*Primary*		1.02 (0.55 – 1.90)	0.99 (0.53 – 1.84)
*Secondary or higher*		1 (reference)	1 (reference)
Maternal occupation			
*Unemployed*		1 (reference)	1 (reference)
*Manual*		1.16 (0.53 – 2.59)	1.24 (0.56 –2.74)
*White collar job*		0.80 (0.35 – 1.86)	0.79 (0.34 – 1.81)
Parity			
*1*		1 (reference)	1 (reference)
*2 – 4*		---	---
*≥ 5*		2.52 (1.01 – 6.28)*	2.58 (1.03 – 6.49)*
Birth interval			
*< 18 months*		3.49 (1.60 – 7.59)**	3.47 (1.60 – 7.57)**
*18 – 36 months*		1.22 (0.77 – 1.93)	1.24 (0.56 – 2.74)
*> 36 months*		1 (reference)	1 (reference)
Breastfeeding			
*Yes*		1 (reference)	1 (reference)
*No*		133.50 (75.89 – 234.83)***	142.31 (80.19 – 252.54)***
Health seeking behaviour			
*Very low (25%)*		1 (reference)	1 (reference)
*Low (26-50%)*		0.21 (0.13 – 0.36)***	0.21 (0.12 – 0.35)***
*Average (50-75%)*		0.26 (0.14 – 0.49)***	0.25 (0.13 – 0.46)***
*High (76-100%)*		---	---
**Paternal factor**			
Paternal occupation			
*Farming*		0.75 (0.37 – 1.57)	0.83 (0.40 – 1.73)
*Manual*		0.95 (0.48 – 1.89)	0.93 (0.46 – 1.85)
*White collar job*		1 (reference)	1 (reference)
Paternal education			
*No education*		0.86 (0.43 – 1.70)	0.96 (0.48 – 1.91)
*Primary*		0.66 (0.29 – 1.50)	0.62 (0.27 – 1.42)
*Secondary or higher*		1 (reference)	1 (reference)
Wealth index			
*Poor*		0.55 (0.30 – 1.01)	0.75 (0.39 – 1.43)
*Middle*		1 (reference)	1 (reference)
*Rich*		1.54 (0.75 – 3.13)	1.32 (0.64 – 2.76)
Ethnicity			
*Akan*		1 (reference)	1 (reference)
*Ga/Guan*		0.77 (0.31 – 1.95)	0.86 (0.34 – 2.18)
*Ewe*		0.73 (0.33 – 1.62)	0.79 (0.35 – 1.76)
*Mole-dagbani*		0.99 (0.49 – 1.99)	1.21 (0.60 – 2.47)
*Grussi/Gruma*		1.22 (0.56 – 2.63)	1.59 (0.71 – 3.57)
*Others*		0.95 (0.35 – 2.59)	1.14 (0.42 – 3.10)
**Community-level determinants**			
Community socio-economic disadvantage			
*Low deprivation*			1 (reference)
*Moderate deprivation*			2.05 (1.03 – 4.07)*
*High deprivation*			3.38 (1.42 – 8.04)**
**Random effect**			
**Area Variance**	0.235*	1.64 × 10^-13^	1.02 × 10^-9^
**MOR**	1.58	1.000000385	1.000030341
**ICC (latent variable method)**	0.07	4.98 × 10^-14^	3.10 × 10^-10^
**AIC**	2281.7392	819.77086	816.31818

Model 1 is the null model, contained no explanatory variable.

Model 2 adjusted for individual-level characteristics.

Model 3 adjusted for both individual-level and community-level characteristics.

*Abbreviations*: *OR* odds ratio 95%, *CI* 95% confidence interval, *MOR* median odds ratio, *ICC* intracluster correlation.

--- denotes estimate omitted by Stata software.

***p < 0.001, **p < 0.01, and *p < 0.05.

Following the decomposition of the neonatal variance in model 1, individual-level covariates were introduced into the empty model to form model 2. It was observed that the community-level variance reduced drastically in model 2. This indicated that the composition of the individual characteristics within the communities explained most of the community-level variance observed in the null model. However, we extended model 2 by introducing community-level covariates to form model 3. Community-level variance was no longer significant after adjusting for both individual and community-level factors. It is important to mention that a random intercept model was constructed rather than the usual single-level model, not only because of the hierarchical nature of the data but also not to have biased associations.

### Fixed effects (Measures of associations)

Table 3 also shows the fixed effects for individual and community-level determinants. Fixed effects of model 2 show the associations between neonatal mortality and individual-level determinants when the community-level covariates were not considered while the fixed effects of model 3 show the associations between neonatal mortality and both individual and community-level determinants. After considering both individual and community-level characteristics in model 3, it was noticed that infants of multiple gestation had a five times higher likelihood of dying before attaining the age of one month (OR 5.30; 95% CI 2.81 – 10.00; P-value < 0.001). Similarly, infants with LBW had a twofold increase in the likelihood of dying during the neonatal period compared to their peers with normal birth weight (OR 2.01; 95% CI 1.23 – 3.30; P-value < 0.01). In addition to child factors that were observed to influence neonatal survival, certain maternal factors were shown to be associated with child survival within the first 28 days of life. Infants delivered by grand multiparous women were 3 times more likely to die compared to those delivered by nulliparous women (OR 2.58; 95% CI 1.03 – 6.49; P-value < 0.05). Long birth spacing and breastfeeding had a protective effect on child survival during the neonatal stage. The likelihood of dying during the neonatal life increased 3.5 fold in infants with birth spacing of < 18 months compared to their peers with more than 36 months (OR 3.47; 95% CI 1.60 – 7.57; P-value < 0.01). Not being breastfed was strongly associated with neonatal mortality (OR 142.31; 95% CI 80.19 –252.54; P-value < 0.001). The utilization of antenatal, delivery and postnatal services by women with good health seeking behaviour reduced the likelihood of losing their babies during neonatal life compared to those with the least favourable health seeking behaviour [(OR 0.21; 95% CI 0.12 – 0.35; P-value < 0.001); (OR 0.25; 95% CI 0.13 – 0.46; P-value < 0.001)]. Infants of mothers that were living in communities with high and moderate socioeconomic disadvantage had a 3.4 and 2 fold increase in likelihood of neonatal death respectively (OR 3.38; 95% CI 1.42 – 8.04; P-value < 0.01; OR 2.05; 95% CI 1.03 – 4.07; P-value < 0.05) compared to those residing in areas with the least socioeconomic disadvantage.

### Model fit statistics

There was a progressive increase in the loglikelihood observed in model 1 when we fitted model 2 and model 3. More importantly the AIC were decreasing from model 1 to 3. This implies that model 3 explained the determinants better than model 1 and 2.

## Discussion

### Main findings

The findings from this study shed light beyond the contribution of individual characteristics to neonatal survival. They demonstrated how the communities where the mothers were living shaped the prevalence of neonatal mortality in conjunction with the composition of the individual characteristics. Both individual and community-level characteristics showed significant associations with neonatal survival. Living in a socioeconomic deprived community (rural with a high prevalence of illiteracy, poverty and unemployment) was inversely associated with neonatal survival. These four components of community socioeconomic deprivation coexist together in varying proportions in the communities and the intensity of deprivation depends on them. Prior studies in LMICs have not adequately examined the association between community-level factors and neonatal mortality even though community-level factors have been shown to be associated with under-five mortality and morbidity [22-24]. There are multifaceted plausible explanations for this association. Dwelling in a rural community where illiteracy, poverty, and unemployment are coexisting will influence neonatal survival via multiple channels. People living in the same community with socioeconomic deprivation tend to be similar in terms of health outcome (neonatal mortality) because of the shared community characteristics which may mediate its impact through poor access to health care services, inability to afford health care costs, poor personal and environmental hygiene, poor nutrition, ignorance of the importance of health care services and more. Community factors will impact their effect on the health outcome (neonatal mortality) through the individual-level factors. Although it is not the aim of this study to explain the underlying mechanism of the observed association, we expect that living in a community with low socioeconomic status will mediate its effect on neonatal survival through individual-level factors based on the results of a previous study [26]. Several neonatal, maternal, antenatal, delivery and postnatal characteristics were shown to be associated with neonatal mortality in the present analysis. Being an infant of multiple gestation was negatively associated with neonatal survival as previously reported in studies from LMICs [21,35]. The plausible explanation for this association is that multiple pregnancies/multiple births have a higher risk of prematurity and small-for-gestational age (SGA). These morbid conditions will make the infants more prone to critical medical complications which might not be adequately managed in low-resource settings. Subsequent adjustment for LBW and other determinants did not alter the observed association between multiple pregnancy and neonatal mortality. In addition, LBW showed an independent association with neonatal mortality which is consistent with medical knowledge and outcomes of previous studies. Maternal factors found to be associated with neonatal mortality were breast feeding and birth spacing. Breast feeding was identified to have the strongest association with neonatal mortality; the odds of this association was observed to be very high (OR = 142.31; 95% CI = 80.19 –252.54), thus it is important to mention that only 3% of the newborns were not breastfed; of these 70% died before attaining the age of one month whereas only 2% of the breastfed babies eventually died in the same period.

Breastfeeding has been reported to have a protective effect against hypothermia and hypoglycaemia which are contributors to neonatal deaths [36]. Failure of the newborns to receive colostrum following delivery will make them more susceptible to infections because of their immure immune system. Results of a prior study conducted in Ghana showed that delayed breastfeeding initiation caused an increase in neonatal mortality through infection related diseases; [18] findings from other population-based studies support this notion [36-38].

Adequate birth spacing was another important maternal factor noticed to have a protective effect on neonatal survival. The length of the birth interval was inversely related to neonatal mortality; suggesting that the longer the mothers waited before having the next pregnancy the better their chance of being recuperated well from maternal depletion associated with the prior pregnancy. This will ensure an adequate supply of essential nutritional support for the growth and well being of a subsequent pregnancy. This is consistent with previous studies [19,35,38,39].

Utilization of antenatal, delivery and postnatal services were inversely related to neonatal mortality. Infants of mothers that utilized these health services were found to have a better neonatal survival. The health of a neonate deteriorates considerably faster than the health of an adult following infection, but neonates also recover very fast if appropriate intervention is received as early as possible. Thus, it is important that mothers seek health intervention promptly in case of illness to save the lives of their infants.

Most of the maternal health indicators that were operationalized to generate maternal health seeking behaviour have been shown to have a similar influence on neonatal mortality. Mothers that possessed good health seeking behaviour such as having tetanus toxoid during pregnancy; and received skilled antenatal, delivery and postnatal care have been shown to reduce the chances of neonatal death among their siblings [19,38,40]. The impact of birth spacing, breast feeding and utilization of antenatal, delivery and postnatal services have clearly demonstrated the possible impact of the continuum-of-care approach [41] from antenatal to postnatal life on the survival and well-being of newborns. Mothers with a good health seeking behaviour will have a better uptake of the components of this approach from antenatal to postnatal care, as they will be more likely to receive tetanus toxoid, breast feeding counselling, birth preparedness, blood supplements, skilled delivery, birth spacing, immediate neonatal care and more.

### Study limitations and strengths

Data explored in this study came from two nationally representative surveys with household and individual response rates of 99% and 96% respectively [16,25]. Recall bias in this type of data has been shown to be low [42,43]; and appropriate statistical methods were applied. However, considering the fact that we used secondary data in this study unobserved confouders might be a problem. A high odds ratio was observed among the 3% of infants that were not breastfed. Some babies may have died so early that not being breastfed would not have contributed to their death; the observed association between breastfeeding and neonatal mortality might have been overestimated. Because only surviving mothers had the opportunity to be interviewed, there is a possibility that neonatal deaths might have been underreported. For instance, mothers that died during labour with their babies due to obstetric complications would be omitted in the current analysis, implying that the burden associated with neonatal mortality might even be larger than presented. As information on early neonatal death was not available, we could not assess the effect of removing early neonatal deaths on the observed association between breastfeeding and neonatal mortality, which may have resulted in an overestimation of the true association between breastfeeding and neonatal mortality. With regard to classification in birth weight categories in the current study, we acknowledge that recall of birth weight size by mothers and subsequent classification in low and normal birth weight might have resulted in some misclassification of exposure and loss of information.

### Recommendations

This study demonstrated that both individual and community characteristics have a substantial impact on child survival in neonatal life. Thus, a comprehensive approach should be taken in combating neonatal mortality. Provision of universal basic education, creation of job opportunities, poverty alleviation, women empowerment programmes, and abridging the inequality gaps between rural and urban areas are important community-based interventions that will alleviate the impacts of community socioeconomic deprivation.

This cannot be achieved without a strong financial and political commitment of government and non-governmental bodies. Earlier reports showed that neonatal mortality has not received adequate financial attention. Even although it accounted for more than 40% of under-five mortality [44], over 50% of infant mortality and up to the total deaths caused by Acquired Immunodeficiency Syndrome (AIDS) and malaria combined [8], yet neonatal mortality has been receiving inadequate financial attention [45].

In addition to the environment in which women live, individual factors such as neonatal, antenatal, delivery and postnatal factors were important determinants of child survival in neonatal life. Small babies (preterm, small for gestational age or both) and infants of multiple gestation had a higher likelihood of dying in the neonatal stage indicating needs to provide essential neonatal care to this vulnerable group of neonates. Health system strengthening is needed in order to provide high quality, affordable and accessible health care for them. Integration of neonatal care to the Integrated Management of Childhood Illness (IMCI) programme will fill the observed gap (first seven days of life) between the Safe Motherhood Initiative (SMI) and IMCI [2,8] and this is a critical period when three-quarters of neonatal mortality occur [4].

Infants born to multiparous mothers with short birth spacing intervals were more likely to die in neonatal life while exclusive breastfeeding was found to have a protective effect on neonatal survival implying the importance of effective implementation of family planning programmes, reproductive health education, use of contraceptives and promotion of exclusive breastfeeding. Maternal health seeking behaviour towards antenatal, delivery and postnatal services plays a vital role in neonatal survival, indicating why decision and policy makers and non-governmental bodies should implement the continuum-of-care approach for maternal and newborn healthcare services spanning from antenatal, to delivery, immediate neonatal and postnatal care. The interagration of neonatal care to the IMCI, use of contraceptive, reproductive health education, exclusive breast feeding and other intervention programs can be delivered through a continuum-of-care approach to ensure continuity of healthcare services for infants and their mothers.

## Conclusion

This study examined nationally representative data on neonatal mortality over a decade by analysing a combined dataset of the 2003 and 2008 Ghana demographic and health surveys. The outcomes of the study demonstrated both community (community socioeconomic disadvantage) and individual (neonatal, maternal, antenatal, delivery and postnatal) level factors to be significantly associated with infant survival within the first 28 days of life. A comprehensive approach comprising community-based interventions (universal basic education, poverty alleviation, women empowerment and infrastructural development) and the continuum-of-care for maternal-newborn healthcare services is needed to reduce the burden of neonatal mortality in LMICs.

## Competing interests

The authors declare that they have no competing interests.

## Authors’ contributions

(GAK) and (KKG) conceived the study idea. (GAK) was responsible for the literature review, data extraction and analysis and wrote the first draft of the manuscript. All authors (GAK), (KKG), (DEG), (EA), (MAC), and (IAA) scientifically reviewed and approved the final version of the manuscript.

## Pre-publication history

The pre-publication history for this paper can be accessed here:

http://www.biomedcentral.com/1471-2393/14/165/prepub

## References

[B1] Lawn JE Cousens S Zupan J 4 million neonatal deaths: When? Where? Why? Lancet 2005 365 9462 891 900 10.1016/S0140-6736(05)71048-5 15752534

[B2] Martines J Paul VK Bhutta ZA Koblinsky M Soucat A Walker N Bahl R Fogstad H Costello A Lancet Neonatal Survival Steering Team Neonatal survival: a call for action Lancet 2005 365 9465 1189 1197 10.1016/S0140-6736(05)71882-1 15794974

[B3] United Nations Millennium Declaration 2012 http://www.un.org/millennium/declaration/ares552e.pdf. (Accessed 13/07/2012)

[B4] Newborns: reducing mortality 2012 http://www.who.int/mediacentre/factsheets/fs333/en/. (Accessed 09/07/2012)

[B5] Committing to child survival: a promise renewed 2014 New York, United States: UNICEF http://www.unicef.org/videoaudio/PDFs/APR_Progress_Report_2012_final.pdf. (Accessed 19/01/2014)

[B6] Darmstadt GL Bhutta ZA Cousens S Adam T Walker N de BL Evidence-based, cost-effective interventions: how many newborn babies can we save? Lancet 2005 365 9463 977 988 10.1016/S0140-6736(05)71088-6 15767001

[B7] Knippenberg R Lawn JE Darmstadt GL Begkoyian G Fogstad H Walelign N Paul VK Lancet Neonatal Survival Steering Team Systematic scaling up of neonatal care in countries Lancet 2005 365 9464 1087 1098 10.1016/S0140-6736(05)71145-4 15781104

[B8] Lawn JE Cousens SN Darmstadt GL Bhutta ZA Martines J Paul V Knippenberg R Fogstad H Lancet Neonatal Survival Series steering team 1 year after The Lancet Neonatal Survival Series–was the call for action heard? Lancet 2006 367 9521 1541 1547 10.1016/S0140-6736(06)68587-5 16679168 PMC7138031

[B9] Oestergaard MZ Inoue M Yoshida S Mahanani WR Gore FM Cousens S Lawn JE Mathers CD United Nations Inter-Agency Group for Child Mortality Estimation and the Child Health Epidemiology Reference Group Neonatal mortality levels for 193 countries in 2009 with trends since 1990: a systematic analysis of progress, projections, and priorities PLoS Med 2011 8 8 e1001080 10.1371/journal.pmed.1001080 21918640 PMC3168874

[B10] The State of the World’s Children Celebrating 20 years of the Convention on the Rights of the Child 2009 New York, United States: UNICEF

[B11] Black RE Cousens S Johnson HL Lawn JE Rudan I Bassani DG Jha P Campbell H Walker CF Cibulskis R Eisele T Liu L Mathers C Child Health Epidemiology Reference Group of WHO and UNICEF Global, regional, and national causes of child mortality in 2008: a systematic analysis Lancet 2010 375 9730 1969 1987 10.1016/S0140-6736(10)60549-1 20466419

[B12] Baiden F Hodgson A Adjuik M Adongo P Ayaga B Binka F Trend and causes of neonatal mortality in the Kassena-Nankana district of northern Ghana, 1995–2002 Trop Med Int Health 2006 11 4 532 539 10.1111/j.1365-3156.2006.01582.x 16553937

[B13] Engmann C Walega P Aborigo RA Adongo P Moyer CA Lavasani L Williams J Bose C Binka F Hodgson A Stillbirths and early neonatal mortality in rural Northern Ghana Trop Med Int Health 2011 17 3 272 282 22175764 10.1111/j.1365-3156.2011.02931.x

[B14] Edmond KM Quigley MA Zandoh C Danso S Hurt C Owusu Agyei S Kirkwood BR Aetiology of stillbirths and neonatal deaths in rural Ghana: implications for health programming in developing countries Paediatr Perinat Epidemiol 2008 22 5 430 437 10.1111/j.1365-3016.2008.00961.x 18782251

[B15] Edmond KM Quigley MA Zandoh C Danso S Hurt C Owusu Agyei S Kirkwood BR Diagnostic accuracy of verbal autopsies in ascertaining the causes of stillbirths and neonatal deaths in rural Ghana Paediatr Perinat Epidemiol 2008 22 5 417 429 10.1111/j.1365-3016.2008.00962.x 18782250

[B16] Ghana demographic and health survey 2008 2009 http://www.measuredhs.com/pubs/pdf/FR221/FR221.pdf. Accessed 17/06/2012)

[B17] Shiffman J Issue attention in global health: the case of newborn survival Lancet 2010 375 9730 2045 2049 10.1016/S0140-6736(10)60710-6 20569844

[B18] Edmond KM Zandoh C Quigley MA Menga-Etego S Owusu-Agyei S Kirkwood BR Delayed breastfeeding initiation increases risk of neonatal mortality Pediatrics 2006 117 3 e380 e386 10.1542/peds.2005-1496 16510618

[B19] Titaley CR Dibley MJ Agho K Roberts CL Hall J Determinants of neonatal mortality in Indonesia BMC Public Health 2008 8 232 10.1186/1471-2458-8-232 18613953 PMC2478684

[B20] Engmann C Matendo R Kinoshita R Ditekemena J Moore J Goldenberg RL Tshefu A Carlo WA McClure EM Bose C Wright LL Stillbirth and early neonatal mortality in rural Central Africa Int J Gynaecol Obstet 2009 105 2 112 117 10.1016/j.ijgo.2008.12.012 19201402 PMC3972762

[B21] Diallo AH Meda N Ouedraogo WT Cousens S Tylleskar T A prospective study on neonatal mortality and its predictors in a rural area in Burkina Faso: can MDG-4 be met by 2015? J Perinatol 2011 31 10 656 663 10.1038/jp.2011.6 21372798 PMC3183235

[B22] Adekanmbi VT Kayode GA Uthman OA Individual and contextual factors associated with childhood stunting in Nigeria: a multilevel analysis Matern Child Nutr 2013 9 2 244 259 10.1111/j.1740-8709.2011.00361.x 22004134 PMC6860873

[B23] Antai D Regional inequalities in under-5 mortality in Nigeria: a population-based analysis of individual- and community-level determinants Popul Health Metr 2011 9 6 10.1186/1478-7954-9-6 21388522 PMC3065413

[B24] Uthman OA A multilevel analysis of individual and community effect on chronic childhood malnutrition in rural Nigeria J Trop Pediatr 2009 55 2 109 115 18845589 10.1093/tropej/fmn093

[B25] Ghana demographic and health survey 2003 2003 http://www.measuredhs.com/pubs/pdf/FR152/FR152.pdf. (Accessed 25/06/2012)

[B26] Mosley WH Chen LC An analytical framework for the study of child survival in developing countries. 1984 Bull World Health Organ 2003 1984 81 2 140 145 PMC2572391 12756980

[B27] Macro ORC Bank W DHS and World Bank use wealth index to measure socioeconomic status DHS Dimensions 2002 4 2 1 2

[B28] Vyas S Kumaranayake L Constructing socio-economic status indices: how to use principal components analysis Health Policy Plan 2006 21 6 459 468 10.1093/heapol/czl029 17030551

[B29] Filmer D Pritchett LH Estimating wealth effects without expenditure data–or tears: an application to educational enrollments in states of India Demography 2001 38 1 115 132 11227840 10.1353/dem.2001.0003

[B30] Major JM Doubeni CA Freedman ND Park Y Lian M Hollenbeck AR Schatzkin A Graubard BI Sinha R Neighborhood socioeconomic deprivation and mortality: NIH-AARP diet and health study PLoS One 2010 5 11 e15538 10.1371/journal.pone.0015538 21124858 PMC2990774

[B31] Turney K Harknett K Neighborhood disadvantage, residential stability, and perceptions of instrumental support among new mothers J Fam Issues 2010 31 4 499 524 10.1177/0192513X09347992 22102766 PMC3217243

[B32] Wight RG Cummings JR Miller-Martinez D Karlamangla AS Seeman TE Aneshensel CS A multilevel analysis of urban neighborhood socioeconomic disadvantage and health in late life Soc Sci Med 2008 66 4 862 872 10.1016/j.socscimed.2007.11.002 18160194 PMC3681874

[B33] Uthman OA Moradi T Lawoko S The independent contribution of individual-, neighbourhood-, and country-level socioeconomic position on attitudes towards intimate partner violence against women in sub-Saharan Africa: a multilevel model of direct and moderating effects Soc Sci Med 2009 68 10 1801 1809 10.1016/j.socscimed.2009.02.045 19303687

[B34] StataSE 11 2011 http://www.stata.com. Accessed 03/10/2012)

[B35] Jahn A Kynast-Wolf G Kouyate B Becher H Multiple pregnancy in rural Burkina Faso: frequency, survival, and use of health services Acta Obstet Gynecol Scand 2006 85 1 26 32 10.1080/00016340500324357 16521676

[B36] Huffman SL Zehner ER Victora C Can improvements in breast-feeding practices reduce neonatal mortality in developing countries? Midwifery 2001 17 2 80 92 10.1054/midw.2001.0253 11399129

[B37] Mullany LC Katz J Li YM Khatry SK LeClerq SC Darmstadt GL Tielsch JM Breast-feeding patterns, time to initiation, and mortality risk among newborns in southern Nepal J Nutr 2008 138 3 599 603 18287373 10.1093/jn/138.3.599 PMC2366167

[B38] Zahid GM Mother’s health-seeking behaviour and childhood mortality in Pakistan Pak Dev Rev 1996 35 4 Pt. 2 719 731 12146446

[B39] Rutstein SO Effects of preceding birth intervals on neonatal, infant and under-five years mortality and nutritional status in developing countries: evidence from the demographic and health surveys Int J Gynaecol Obstet 2005 89 Suppl 1 S7 S24 15820369 10.1016/j.ijgo.2004.11.012

[B40] Feng XL Guo S Hipgrave D Zhu J Zhang L Song L Yang Q Guo Y Ronsmans C China’s facility-based birth strategy and neonatal mortality: a population-based epidemiological study Lancet 2011 378 9801 1493 1500 10.1016/S0140-6736(11)61096-9 21924764

[B41] Tinker A ten Hoope-Bender P Azfar S Bustreo F Bell R A continuum of care to save newborn lives Lancet 2005 365 9462 822 825 10.1016/S0140-6736(05)71016-3 15752509

[B42] Hall S Neonatal mortality in developing countries: what can we learn from dhs data? 2005 http://eprints.soton.ac.uk/14214/1/14214-01.pdf (14/11/2012)

[B43] Hill K Choi Y Neonatal mortality in the developing world Demogr Res 2006 14 18 429 452

[B44] The state of the world’s children New York 2009 Report: UNICEF

[B45] Bryce J Victora CG Child survival: countdown to 2015 Lancet 2005 365 9478 2153 2154 10.1016/S0140-6736(05)66752-9 15978906

